# Quartz Crystal Microbalance Platform for SARS-CoV-2 Immuno-Diagnostics

**DOI:** 10.3390/ijms242316705

**Published:** 2023-11-24

**Authors:** Per H. Nilsson, Mahmoud Al-Majdoub, Ahmed Ibrahim, Obaidullah Aseel, Subramanian Suriyanarayanan, Linnea Andersson, Samir Fostock, Teodor Aastrup, Ivar Tjernberg, Ingvar Rydén, Ian A. Nicholls

**Affiliations:** 1 Linnaeus University Centre for Biomaterials Chemistry, Department of Chemistry and Biomedical Sciences, Linnaeus University, SE-39182 Kalmar, Sweden; per.h.nilsson@lnu.se (P.H.N.); esusu@lnu.se (S.S.); linnea.andersson@lnu.se (L.A.); 2 Department of Immunology, University of Oslo and Oslo University Hospital Rikshospitalet, Sognsvannsveien 20, NO-0372 Oslo, Norway; 3 Attana AB, Greta Arwidssons Väg 21, SE-11419 Stockholm, Sweden; mahmoud.almajdoub@attana.com (M.A.-M.); ahmed.ibrahim@attana.com (A.I.); samir.fostock@attana.com (S.F.); teodor.aastrup@attana.com (T.A.); 4 Medical Programme, Faculty of Medicine and Health Sciences, Linköping University, SE-58225 Linköping, Sweden; obaas415@student.liu.se; 5 Department of Clinical Chemistry and Transfusion Medicine, Region Kalmar County, SE-39185 Kalmar, Sweden; ivar.tjernberg@regionkalmar.se; 6 Department of Biomedical and Clinical Sciences, Division of Inflammation and Infection, Linköping University, SE-58183 Linköping, Sweden; 7 Department of Research, Region Kalmar County, SE-39185 Kalmar, Sweden; ingvar.ryden@regionkalmar.se; 8 Department of Biomedical and Clinical Sciences, Division of Clinical Chemistry and Pharmacology, Linköping University, SE-58183 Linköping, Sweden

**Keywords:** chemiluminescence, COVID-19, electrochemiluminescence, quartz crystal microbalance, SARS-CoV-2

## Abstract

Rapid and accurate serological analysis of SARS-CoV-2 antibodies is important for assessing immune protection from vaccination or infection of individuals and for projecting virus spread within a population. The quartz crystal microbalance (QCM) is a label-free flow-based sensor platform that offers an opportunity to detect the binding of a fluid-phase ligand to an immobilized target molecule in real time. A QCM-based assay was developed for the detection of SARS-CoV-2 antibody binding and evaluated for assay reproducibility. The assay was cross-compared to the Roche electrochemiluminescence assay (ECLIA) Elecsys^®^ Anti-SARS-CoV-2 serology test kit and YHLO’s chemiluminescence immunoassay (CLIA). The day-to-day reproducibility of the assay had a correlation of r^2^ = 0.99, *p* < 0.001. The assay linearity was r^2^ = 0.96, *p* < 0.001, for dilution in both serum and buffer. In the cross-comparison analysis of 119 human serum samples, 59 were positive in the Roche, 52 in the YHLO, and 48 in the QCM immunoassay. Despite differences in the detection method and antigen used for antibody capture, there was good coherence between the assays, 80–100% for positive and 96–100% for negative test results. In summation, the QCM-based SARS-CoV-2 IgG immunoassay showed high reproducibility and linearity, along with good coherence with the ELISA-based assays. Still, factors including antibody titer and antigen-binding affinity may differentially affect the various assays’ responses.

## 1. Introduction

The emergence of the SARS-CoV-2 virus and the subsequent COVID-19 pandemic has driven the need to quickly establish rapid, sensitive, and robust diagnostic tools for informing clinicians and policymakers and for vaccine development [1]. Antigen tests to determine the presence of the virus in individuals [2,3] and in the environment [4], together with serological tests to assess the immune response after exposure to the virus or vaccination, are tools that were central for guiding society’s response to the pandemic. These methods have contributed directly to patient care and underlying pathogenesis [5,6], our understanding of SARS-CoV-2 ecology and evolution [7], assessments of its spread and mutational frequency [8], and the impact of measures used to limit the spread of the virus, e.g., by vaccination [9].

Diagnostics providing insight concerning individual immune responses to SARS-CoV-2 infection by quantifying circulating antibodies are important in both the clinical context and for evaluating the broader impact of vaccination programs and mapping the spread of the virus [10]. A variety of detection principles have been used, most commonly antibody-targeting ELISA-based methods [11], as exemplified by the electrochemiluminescence assay (ECLIA) Elecsys^®^ Anti-SARS-CoV-2 serology test kit developed by Roche [12] and YHLO’s chemiluminescence immunoassay (CLIA) [13], where the Roche platform employs the nucleocapsid (N)-antigen, the YHLO assay, and a combination of N- and spike protein (S)-antigens.

Quartz crystal microbalance (QCM) technology [14,15] has been exploited in the development of sensor platforms for use in a wide variety of applications, ranging from environmental monitoring [16], food quality control [17], and drug candidate screening [18,19] to the study of protein–surface interactions [20] and clinical diagnostics [21,22]. Changes in the vibrational frequency of gold electrode-coated quartz crystals allow for the highly sensitive determination of mass bound to the surface, as described by the Sauerbrey equation (Equation (1)) [23]:

(1) $$\Delta f=\frac{-2f_{0}^{2}\Delta m}{A\sqrt{\mu_{q}\rho_{q}}}=C\Delta m$$

where Δ*f* is the change in frequency (Hz), *f*_0_ is the intrinsic resonant frequency of the crystal (Hz), Δ*m* is the mass change (g), *A* is the piezoelectric area of the crystal (cm^2^), *μ_q_* is the shear modulus of quartz (2.95 × 10^11^ g⋅cm^−1^⋅s^−2^), *ρ_q_* is the density of quartz (2.65 g⋅cm^−3^), and *C* is the mass-sensitivity constant (Hz⋅g^−1^).

A feature making this piezoelectric effect-based method particularly appealing is that it offers the potential for developing label-free flow-based diagnostics with real-time detection, a dimension that can provide insights into the kinetics of antibody–antigen interactions [24,25,26]. Accordingly, the objective of the present study was to investigate the possibility to use S-antigen–antibody capture in a QCM platform for developing a robust assay, and to compare this strategy with current platforms based upon different detection techniques and antibody targets. While the principle of using the QCM platform for serologic analysis has been reported [27,28,29], this is, to the best of our knowledge, the first study that systematically evaluates sera for antibodies towards SARS-CoV-2 in a diagnostic context.

## 2. Results

**Figure 1:**
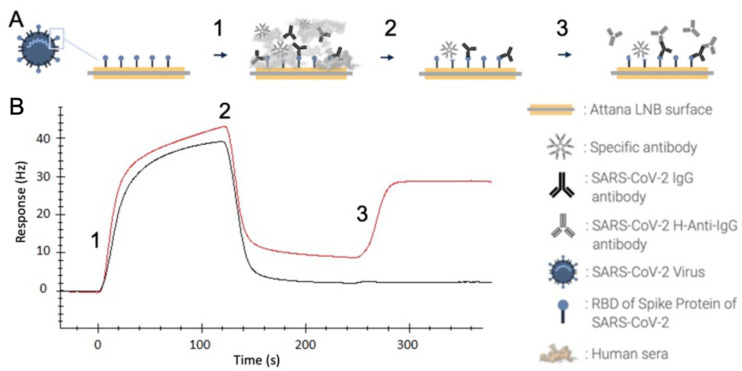
SARS-CoV-2 IgG QCM-based immunoassay. (**A**) Schematic representation of assay procedure. The receptor-binding domain (RBD) of the SARS-CoV-2 spike protein is immobilized on an Attana low non-specific binding sensor chip. Human serum is injected (1) and allowed to interact with the immobilized RBD for 120 s, followed by buffer injection for 127 s (2) where only strongly bound antibodies continue to interact. Anti-human-IgG antibodies are injected (3) for the detection of bound IgG antibodies. (**B**) Sensorgram displaying the response in Hertz over time in relation to the events in panel A. The figure shows two samples with varying titers of SARS-CoV-2 anti-IgG.

**Figure 2:**
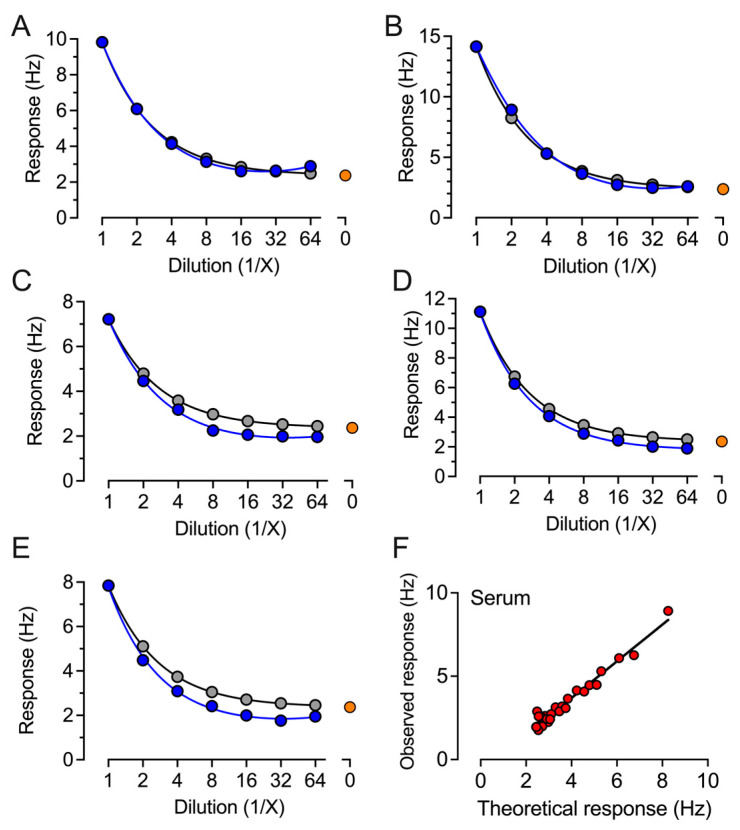
Observed and theoretical response in the QCM-based IgG assay of five samples serially diluted in a negative serum pool. (**A**–**E**) Five randomly selected serum samples were analyzed for antibodies against SARS-CoV-2 spike protein. Each sample was analyzed as undiluted and in two-fold dilutions in a pool of serum from donors negative for SARS-CoV-2 antibodies, plotted as the observed response (blue symbols). The expected response (grey symbols) represents the response for the undiluted sample with the negative serum pool response (orange symbol) subtracted and related to sample dilution. (**F**) All data points from the five randomly selected serum samples were plotted as the observed response to the theoretical response for correlation analysis.

**Figure 3:**
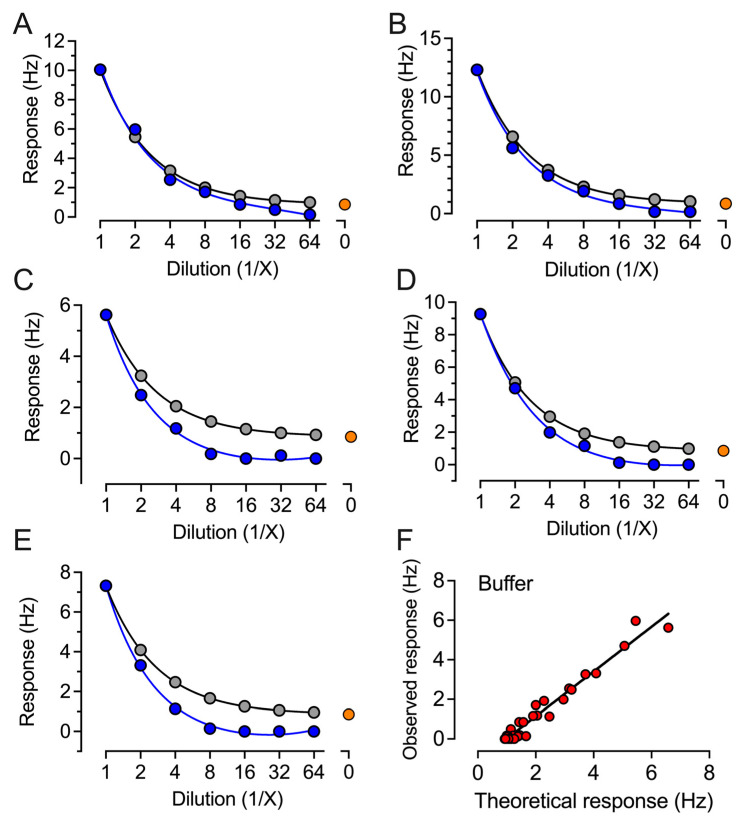
Observed and theoretical response in the QCM-based IgG assay of five samples serially diluted in PBS. (**A**–**E**) Five randomly selected serum samples were analyzed for antibodies against SARS-CoV-2 spike protein. Each sample was analyzed as undiluted and in twofold dilutions in PBS (buffer), plotted as the observed response (blue symbols). The expected response (grey symbols) represents the response for the undiluted sample with buffer response (orange symbol) subtracted and related to sample dilution. (**F**) All data points from the five randomly selected serum samples were plotted as the observed response to the theoretical response for correlation analysis.

The CE-marked ECLIA-based Roche, CLIA-based YHLO, and QCM-based Attana platforms are each based upon different physical principles for the detection of individual immune response to the SARS-CoV-2 virus spike protein or vaccination. The ELISA-based Roche [12] and YHLO assays have been previously reported [13]. The QCM-based Attana platform (Figure 1) was developed using S-antigen immobilized on sensor chips. The change in resonant frequency upon immobilization corresponded to 3.39 × 10^12^ molecules per cm^2^, and no significant changes were observed in the case of control chips. XPS demonstrated the presence of nitrogen when comparing the original chip and S-antigen-derivatized chip (see Figure S2). The assay was initially validated for linearity and reproducibility. Five randomly selected positive samples were serially diluted in serum (Figure 2) or buffer (Figure 3).

**Figure 4:**
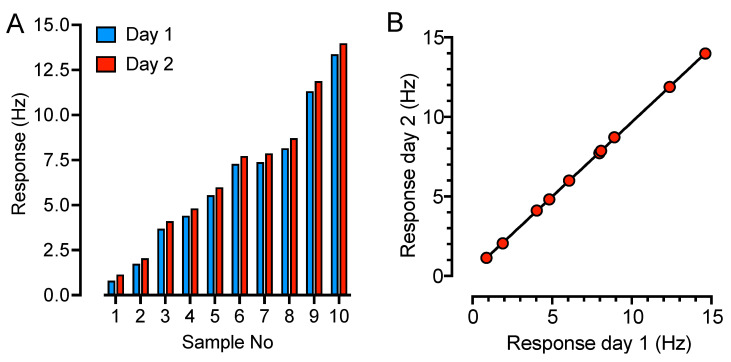
QCM-based SARS-CoV-2 IgG immunoassay reproducibility test. The SARS-CoV-2 IgG-response of ten randomly selected serum samples were tested on two separate SARS-CoV-2 IgG immunoassay chips on two consecutive days. The response (Hz) is plotted in a (**A**) bar graph, at day 1 (blue) and day 2 (red), and (**B**) in an XY-graph for correlation analysis.

The observed response in two-fold dilutions up to 1:64 was compared to the theoretically expected response, expressed as the response in undiluted serum related to the dilution factor. All five samples showed a low deviation between the observed and expected response (Figure 2 and Figure 3); the correlation factors for both dilution in serum and buffer samples were r^2^ = 0.96 (Figure 2F and Figure 3F), indicating good robustness for sample dilution in both serum and buffer. The reproducibility was tested on two consecutive days on two different chips on ten randomly selected samples (Figure 4A). The average response difference was calculated to 5.3%, and the correlation was r^2^ = 0.99 (Figure 4B).

In addition to physical principles for detection, the platforms also differ through their targeting of either one of or both the N- and S-antigens, the impact of which was deemed a parameter important to consider in the comparison of data arising from the three platforms. To compare these platforms, a cross-platform comparative study was undertaken. From an initial series of 335 donor samples analyzed on the Roche-derived ECLIA assay [12], all 59 positive and 60 randomly selected negative samples were then subjected to the CLIA-based YHLO [30] and Attana platform-based QCM assays.

From the collective data, comparable numbers of positive IgG responses were observed with the QCM (*n* = 48), YHLO (*n* = 52), and Roche (*n* = 59) assays (Table 1 and Figures S3 and S4). Of the 48 samples positive in the case of the QCM assay, 45 were also positive in the YHLO assay and 47 in the Roche assay. Of the 59 samples positive in the Roche assay, 52 were positive in the YHLO assay. The corresponding numbers for the 71 negative samples in the QCM assay were 64 in the YHLO assay and 59 for the Roche. Of the 60 negative samples in the Roche assay, all 60 were also negative when interrogated using the YHLO platform.

**Figure 5:**
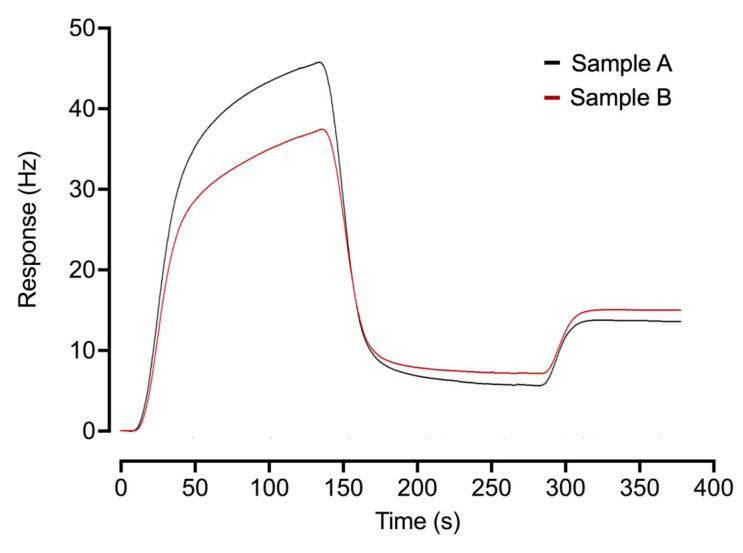
Differences in kinetics of antibodies against SARS-CoV-2 RBD spike protein. Two COVID-19-positive (≥2.5 Hz) donor samples with very similar anti-IgG titer results though with weaker binding kinetics in the case of Sample A, as seen over the interval 180 to 270 s.

The QCM sensorgrams provide access to information concerning the kinetics of the patient IgG response to the SARS-CoV-2 RBD spike protein, as illustrated by two additional COVID-19-positive cases with very similar anti-IgG titer results (7.70 Hz (sample 1) vs. 7.66 Hz (sample 2)) (Figure 5); sample A has weaker binding kinetics, as seen in the reduction in signal from 180 to 270 s arising from IgG dissociation from the target RBD.

## 3. Discussion

The COVID-19 pandemic has driven the development of new diagnostics for determining individual immune responses to infection by variants of the SARS-CoV-2 virus and vaccination. The QCM-based assay was here compared with the ECLIA-based Roche and CLIA-based YHLO-assays with respect to their performance when challenged with positive and negative samples derived from a cohort of 119 donors. Despite being based on different physical principles and using different configurations of the N- and S-antigens, the degree of cohesion between the three platforms over the samples studied was ≥80%. Still, some single samples, including samples 57, 62, and 72 (Table S1), showed strong responses in the Roche and YHLO assays but were negative in the QCM assay. This could be explained by the different immune response towards the S- and N-antigen between individuals [31,32], which can motivate the use of more than one antigen in serological assays. The respective assay platforms each offer potential advantages. The inherent high sensitivity of (electro)chemiluminescence, as deployed in the YHLO CLIA and Roche ECLIA platforms, could provide access to even lower levels of antibody–antigen interaction detection. Both assays involve an initial patient serum incubation with SARS-CoV-2 antigens before a detection step, giving an endpoint measurement. The label-free flow system QCM-based platform can provide direct access to antibody–antigen interaction kinetics data, as the assay response depends on the concentration and binding affinity of the antibodies towards their target antigen, as illustrated by two positive cases, where differences in off-rates are significant (Figure 5). The label-free QCM assay favors antibodies with higher affinity since the interaction time with its immobilized antigen is shorter and observed in a continuous flow, which also allows the assay to be used to provide access to kinetics data [24,33]. Accordingly, a low-antibody titer will result in a low response in the Roche and YHLO assays, yet if these antibodies have a good kinetic profile, the QCM response will be relatively higher, and vice versa. Access to such data can open its use in vaccine screening/development [34]. Further adaption and application of these diagnostic platforms with respect to new mutants of the SARS-CoV-2 virus and for characterizing vaccine-derived impacts on the immune response are a challenge being addressed by us and others.

## 4. Materials and Methods

### 4.1. Blood Samples

Initially, serum samples (*n* = 335) were collected to assess seroprevalence in healthcare workers in Kalmar County, Sweden. None of the donors had been vaccinated against SARS-CoV-2. All samples were screened for SARS-CoV-2 antibodies using the Roche ECLIA-based assay. From the 335 samples, 59 samples tested positive and 276 negative. All positive samples (*n* = 59) were together with 60 randomly selected negative samples subjected for the YHLO and QCM assays (see Table S1).

### 4.2. QCM-Based SARS-CoV-2 IgG Immunoassay

The serum samples were tested for anti-SARS-CoV-2 antibodies using S-antigen derivatization via the quartz resonator and Attana Cell^TM^ 200 biosensor (Figure 1). The assay is designed to quantitatively measure IgG antibodies against S-antigens of SARS-CoV-2 in human serum or plasma.

The receptor-binding domain of the SARS-CoV-2 S-antigen (RBD-Wuhan, Expres2ion biotechnologies, Hørsholm, Denmark) was immobilized onto an Attana low non-specific binding sensor chip (Attana AB, Stockholm, Sweden) using amine coupling according to the manufacturer’s protocol (see Figure S1). Control chips were prepared identically, though in the absence of the S-antigen. Verification of protein immobilization was determined using the Sauerbrey equation and XPS (Figure S2). A 20 μL serum sample was injected at a flow rate of 10 μL/min, interacting with the target for 120 s under continuous flow followed by buffer for 127 s. An anti-human-IgG antibody (20 μg/mL, Medix Biochemica, Rotherham, UK) was subsequently injected for 30 s, followed by buffer for 100 s, and the change in frequency was recorded for a total of 120 s from the injection of the anti-human-IgG antibody. The signal was recorded as the mass change in the sensor surface, which correlates to the amount of IgG antibodies present in the sample. Chip regeneration was performed using a glycine buffer (10 mM, pH 1.0, volume 12.5 μL, flow rate 10 μL/min), with each chip being used for at least 30 studies. Linearity tests were performed on five randomly selected positive samples. The samples were serially diluted up to 1:64 in either a negative serum pool or PBS. A reproducibility test was performed by evaluating the response of ten undiluted randomly selected serum samples on two consecutive days on two individual chips. The total assay time was 8 min, and cut-off values were pre-defined by the manufacturer: ≥2.5 Hz were considered as positive and <2.5 Hz as negative.

### 4.3. ROCHE—Electrochemiluminescence Assay

Serum samples were evaluated for antibodies using the Elecsys^®^ Anti-SARS-CoV-2 serology test kit with the Roche ECLIA platform. The assay is designed to measure total antibodies (IgA, IgM, and IgG) against the N-antigen of SARS-CoV-2 in human serum or plasma. The assay was performed according to the manufacturer’s protocol [12]. Briefly, serum was incubated for 9 min with biotinylated and ruthenylated nucleocapsid antigens, followed by incubation for 9 min with streptavidin-coated magnetic microparticles. The microparticles were captured on the surface of the electrode by magnetic force. Then, unbound substances were removed, and the electrochemiluminescence was induced by applying a voltage that produces the signal proportional to the amount of antibodies present in the serum sample. The cut-off values were pre-defined by the manufacturer: ≥1.0 U/mL was considered as positive and <1.0 U/mL as negative.

### 4.4. YHLO Chemiluminescence Immunoassay

Serum samples were tested using the YHLO iFlash-SARS-CoV-2 IgG with YHLO’s iFlash immunoassay analyzer. The assay is designed for quantitative measurement of IgG antibodies against a combination of N- and S-antigens of SARS-CoV-2 in human serum or plasma. The assay was performed according to the manufacturer’s prescribed protocol [30]. Briefly, serum was incubated with SARS-CoV-2 antigen-coated paramagnetic microparticles, followed by the addition of acridinium-labeled anti-human IgG antibody, which produces a light signal directly proportional to the number of antibodies detected in the sample. The total assay time was 45 min, and the cut-off values were pre-defined by the manufacturer: ≥10 U/mL was considered as positive and <10 U/mL as negative.

## Figures and Tables

**Table 1:** Coherence between QCM, Roche, and YHLO SARS-CoV-2 antibody assays of 119 serum samples ^#.^.

		QCM		Roche		YHLO	
		Positive	Negative	Positive	Negative	Positive	Negative
QCM	Positive	-	-	47	1	45	3
	Negative	-	-	12	59	7	64
Roche	Positive	47	12	-	-	52	7
	Negative	1	59	-	-	0	60
YHLO	Positive	45	7	52	0	-	-
	Negative	3	64	7	60	-	-

^#^ Sera were analyzed for the presence of antibodies against SARS-CoV-2 RBD spike protein. Of the 119 analyzed samples, 48, 52, and 59 were positive in the QCM, Roche, and YHLO assays, respectively.

## Data Availability

Data are contained within the article and Supplementary Materials.

## Supplementary Materials

The supporting information can be downloaded at: https://www.mdpi.com/article/10.3390/ijms242316705/s1.
