# Supplementary material for: Quartz Crystal Microbalance Platform for SARS-CoV-2 Immuno-Diagnostics
Source: Int J Mol Sci. 2023 Nov 24;24(23):16705. doi: 10.3390/ijms242316705 (PMC10705953; doi:10.3390/ijms242316705)
Supplement: Supplementary file 1 [file ijms-24-16705-s001.zip › ijms-2614922-supplementary.pdf]

Supplementary Materials:

Quartz Crystal Microbalance Platform for SARS-CoV-2 Immuno-diagnostics

Per H. Nilsson [1,2], Mahmoud Al-Majdoub [3], Ahmed Ibrahim [3], Obaidullah Aseel [4], Subramanian Suriyanarayanan [1], Linnea Andersson [1], Samir Fostock [3], Teodor Aastrup [3], Ivar Tjernberg [5], Ingvar Rydén [6], and Ian A. Nicholls [1*]

[1] Linnaeus University Centre for Biomaterials Chemistry, Department of Chemistry and Biomedical Sciences, Linnaeus University, SE-39182 Kalmar, Sweden
[2] Department of Immunology, University of Oslo and Oslo University Hospital Rikshospitalet, Sognsvannsveien 20, NO-0372 Oslo, Norway
[3] Attana AB, Greta Arwidssons Väg 21, SE-11419 Stockholm, Sweden
[4] Medical Programme, Faculty of Medicine and Health Sciences, SE-58225 Linköping University, Sweden
[5] Department of Clinical Chemistry and Transfusion Medicine, Region Kalmar County, SE-39185 Kalmar, Sweden, and Department of Biomedical and Clinical Sciences, Division of Inflammation and Infection, Linköping University, SE-58183 Linköping, Sweden
[6] Department of Research, Region Kalmar County, SE-39185 Kalmar, Sweden, and Department of Biomedical and Clinical Sciences, Division of Clinical Chemistry and Pharmacology, Linköping University, SE-58183 Linköping, Sweden
* Correspondence: ian.nicholls@lnu.se

Table of Contents

Page	Content
S2	**Fig. S-1**. QCM trace (curve *a*) showing the immobilization of RBD on the LNB chip. (A) activation of LNB-chip by EDC/Sulfo-NHS, (B) RBD immobilization and (C) deactivation with ethanolamine. Curve *b* is the corresponding reference LNB chip without RBD.
S3	**Fig. S-2**. Binding energy profile recorded with XPS for the LNB chip measured (A) before and (B) after the immobilization of RBD
S4	**Fig. S-3.** Injection of serum sample on reference chip (without immobilized RBD).
S5	**Table S-1**. Raw data from the detection of SARS-CoV-2 antibodies in 119 serum samples analyzed by the QCM, YHLO, and Roche assays.
S8	**Fig. S-4**. Coherence between QCM, Roche, and YHLO SARS-CoV-2 antibody assays – graphical representation

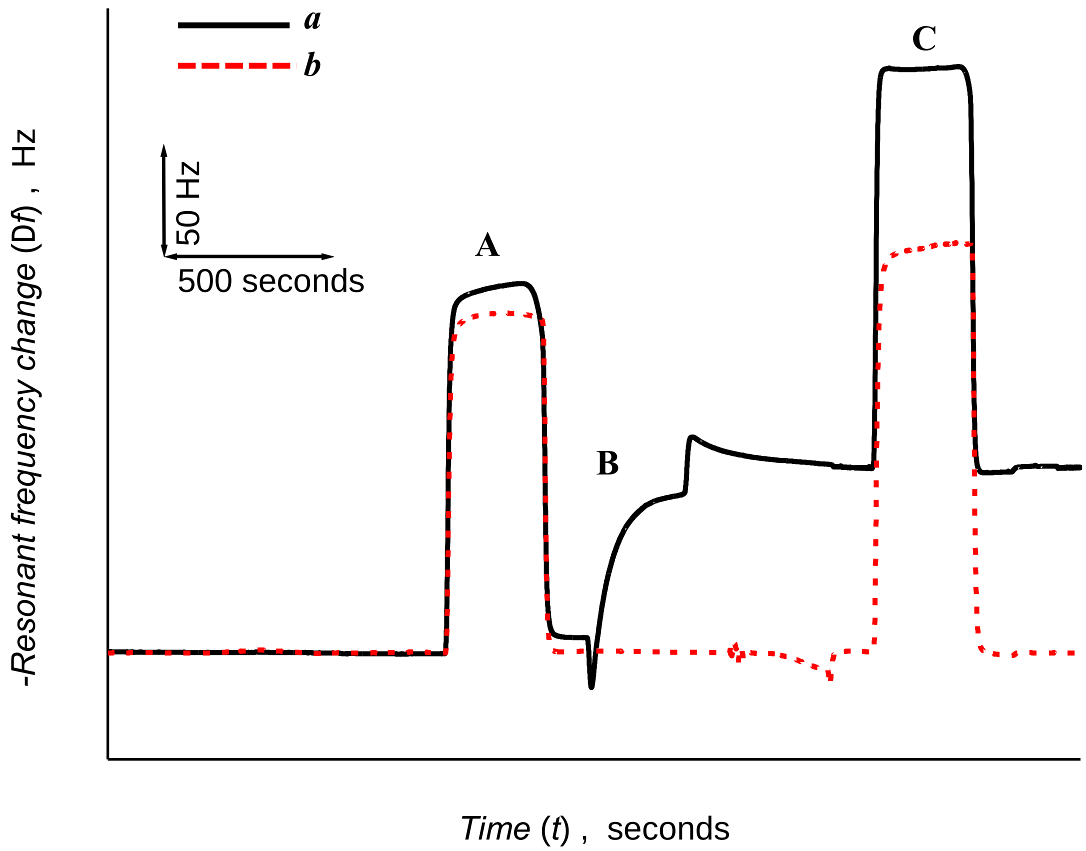

Fig. S-1. QCM trace (curve *a*) showing the immobilization of RBD on the LNB chip. (A) activation of LNB-chip by EDC/Sulfo-NHS, (B) RBD immobilization and (C) deactivation with ethanolamine. Curve *b* is the corresponding reference LNB chip without RBD.

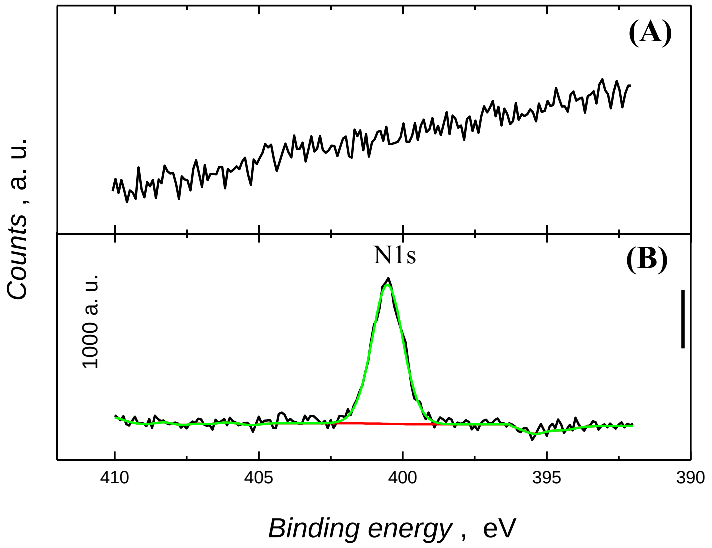

Fig. S-2. Binding energy profile recorded with XPS for the LNB chip measured (A) before and (B) after the immobilization of RBD. The presence of nitrogen after immobilization (together with antibody-based QCM response) provides evidence for the presence of the protein on the sensor chip surface.

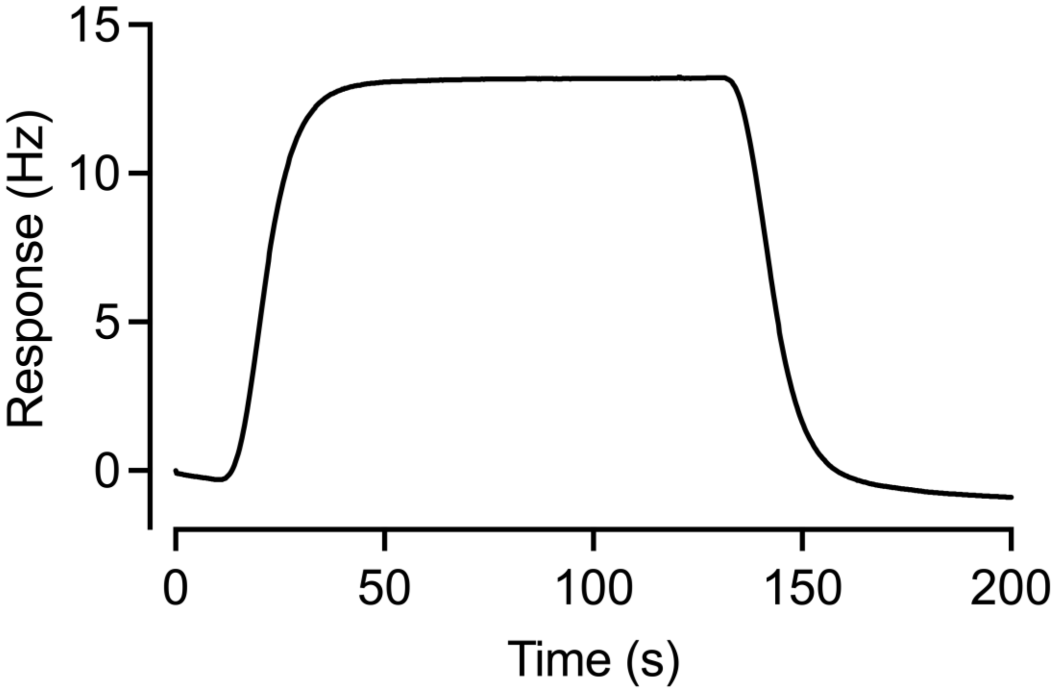

Fig. S-3. Injection of serum sample on reference chip (without immobilized RBD).

Table SI-1. Raw data for the detection of SARS-CoV-2 antibodies in 119 serum samples analyzed by the QCM, YHLO, and Roche assays. A response ≥2.5 in the QCM assay, ≥10.00 in the YHLO, and ≥1.00 in the Roche assay was regarded as a positive response (marked in green), and a response below was regarded as negative (marked in red). The samples are sorted according to the response in the QCM assay.

	QCM		YHLO		Roche	
**Sample**	**Response (Hz)**	**≥2.50**	**Response (U/mL)**	**≥10.00**	**Response (U/mL)**	**≥1.00**
**1**	14.09	Pos	46.27	Pos	71.1	Pos
**2**	14.08	Pos	36.53	Pos	32.0	Pos
**3**	13.98	Pos	23.78	Pos	17.70	Pos
**4**	13.56	Pos	105.03	Pos	78.90	Pos
**5**	13.45	Pos	96.33	Pos	86.20	Pos
**6**	11.88	Pos	86.80	Pos	151	Pos
**7**	9.85	Pos	52.65	Pos	89.6	Pos
**8**	9.72	Pos	105.65	Pos	110	Pos
**9**	8.74	Pos	103.65	Pos	142	Pos
**10**	8.72	Pos	27.68	Pos	50.3	Pos
**11**	8.44	Pos	7.99	Neg	6.93	Pos
**12**	8.38	Pos	111.45	Pos	137	Pos
**13**	7.87	Pos	12.24	Pos	25.7	Pos
**14**	7.72	Pos	62.62	Pos	110	Pos
**15**	7.44	Pos	129.18	Pos	116	Pos
**16**	7.33	Pos	65.07	Pos	38.8	Pos
**17**	7.17	Pos	52.89	Pos	127	Pos
**18**	6.53	Pos	29.99	Pos	52.9	Pos
**19**	6.31	Pos	103.04	Pos	58.9	Pos
**20**	6.31	Pos	81.19	Pos	168	Pos
**21**	5.99	Pos	87.07	Pos	108	Pos
**22**	5.96	Pos	13.19	Pos	31.6	Pos
**23**	5.79	Pos	129.48	Pos	90.7	Pos
**24**	5.73	Pos	67.62	Pos	92.0	Pos
**25**	5.68	Pos	21.54	Pos	47.6	Pos
**26**	5.18	Pos	100.25	Pos	111	Pos
**27**	4.94	Pos	83.87	Pos	146	Pos
**28**	4.81	Pos	15.96	Pos	13.6	Pos
**29**	4.69	Pos	85.28	Pos	158	Pos
**30**	4.67	Pos	44.01	Pos	87.4	Pos
**31**	4.63	Pos	24.04	Pos	30.0	Pos
**32**	4.60	Pos	24.62	Pos	45.4	Pos
**33**	4.43	Pos	20.57	Pos	37.0	Pos
**34**	4.20	Pos	26.94	Pos	55.6	Pos
**35**	4.14	Pos	64.08	Pos	141	Pos
**36**	4.11	Pos	63.34	Pos	152	Pos
**37**	4.08	Pos	60.36	Pos	125	Pos

**38**	4.03	Pos	25.77	Pos	20.1	Pos
**39**	4.03	Pos	49.39	Pos	79.9	Pos
**40**	3.97	Pos	45.48	Pos	117	Pos
**41**	3.36	Pos	39.15	Pos	98.3	Pos
**42**	3.21	Pos	18.18	Pos	22.1	Pos
**43**	3.17	Pos	132.27	Pos	106	Pos
**44**	2.78	Pos	60.23	Pos	146	Pos
**45**	2.60	Pos	64.37	Pos	149	Pos
**46**	2.54	Pos	20.48	Pos	44.0	Pos
**47**	2.54	Pos	0.08	Neg	0.08	Neg
**48**	2.52	Pos	3.27	Neg	2.24	Pos
**49**	2.46	Neg	0.50	Neg	0.08	Neg
**50**	2.41	Neg	28.45	Pos	54.2	Pos
**51**	2.17	Neg	0.12	Neg	0.08	Neg
**52**	2.14	Neg	5.80	Neg	1.98	Pos
**53**	2.05	Neg	12.31	Pos	28.6	Pos
**54**	1.96	Neg	0.53	Neg	0.09	Neg
**55**	1.94	Neg	4.24	Neg	6.45	Pos
**56**	1.89	Neg	0.10	Neg	0.08	Neg
**57**	1.84	Neg	123.72	Pos	123	Pos
**58**	1.74	Neg	6.54	Neg	6.41	Pos
**59**	1.71	Neg	3.04	Neg	4.19	Pos
**60**	1.68	Neg	0.09	Neg	0.09	Neg
**61**	1.44	Neg	0.09	Neg	0.08	Neg
**62**	1.43	Neg	70.79	Pos	149	Pos
**63**	1.42	Neg	0.17	Neg	0.09	Neg
**64**	1.40	Neg	0.55	Neg	0.08	Neg
**65**	1.36	Neg	0.10	Neg	0.08	Neg
**66**	1.28	Neg	13.45	Pos	9.33	Pos
**67**	1.20	Neg	0.14	Neg	0.08	Neg
**68**	1.20	Neg	0.14	Neg	0.09	Neg
**69**	1.19	Neg	0.14	Neg	0.09	Neg
**70**	1.18	Neg	5.02	Neg	0.30	Neg
**71**	1.17	Neg	0.11	Neg	0.08	Neg
**72**	1.14	Neg	24.75	Pos	51.7	Pos
**73**	1.10	Neg	0.17	Neg	0.09	Neg
**74**	1.08	Neg	0.10	Neg	0.08	Neg
**75**	1.00	Neg	0.23	Neg	0.09	Neg
**76**	0.98	Neg	0.12	Neg	0.09	Neg
**77**	0.97	Neg	0.59	Neg	0.10	Neg
**78**	0.96	Neg	0.78	Neg	0.09	Neg
**79**	0.88	Neg	13.89	Pos	18.0	Pos
**80**	0.87	Neg	0.17	Neg	0.10	Neg

**81**	0.85	Neg	0.20	Neg	1.29	Pos
**82**	0.81	Neg	0.12	Neg	0.08	Neg
**83**	0.79	Neg	0.12	Neg	0.08	Neg
**84**	0.74	Neg	0.28	Neg	0.09	Neg
**85**	0.70	Neg	0.17	Neg	0.08	Neg
**86**	0.70	Neg	2.94	Neg	0.93	Neg
**87**	0.66	Neg	0.37	Neg	0.08	Neg
**88**	0.65	Neg	0.33	Neg	0.09	Neg
**89**	0.62	Neg	0.52	Neg	0.08	Neg
**90**	0.59	Neg	0.30	Neg	0.08	Neg
**91**	0.55	Neg	0.15	Neg	0.08	Neg
**92**	0.52	Neg	0.52	Neg	0.09	Neg
**93**	0.50	Neg	0.26	Neg	0.09	Neg
**94**	0.50	Neg	0.17	Neg	0.09	Neg
**95**	0.46	Neg	0.44	Neg	0.09	Neg
**96**	0.42	Neg	0.65	Neg	0.08	Neg
**97**	0.40	Neg	0.35	Neg	0.09	Neg
**98**	0.39	Neg	0.88	Neg	0.09	Neg
**99**	0.39	Neg	0.34	Neg	0.09	Neg
**100**	0.35	Neg	0.57	Neg	0.08	Neg
**101**	0.31	Neg	1.60	Neg	0.09	Neg
**102**	0.29	Neg	0.29	Neg	0.09	Neg
**103**	0.29	Neg	1.02	Neg	0.09	Neg
**104**	0.27	Neg	0.72	Neg	0.08	Neg
**105**	0.25	Neg	0.72	Neg	0.08	Neg
**106**	0.15	Neg	0.23	Neg	0.08	Neg
**107**	0.15	Neg	0.23	Neg	0.09	Neg
**108**	0.15	Neg	6.04	Neg	0.09	Neg
**109**	0.13	Neg	0.66	Neg	0.09	Neg
**110**	0.11	Neg	0.30	Neg	0.08	Neg
**111**	0.04	Neg	0.39	Neg	0.08	Neg
**112**	-0.02	Neg	0.24	Neg	0.08	Neg
**113**	-0.05	Neg	1.13	Neg	0.08	Neg
**114**	-0.05	Neg	0.28	Neg	0.09	Neg
**115**	-0.07	Neg	0.21	Neg	0.08	Neg
**116**	-0.10	Neg	0.37	Neg	0.10	Neg
**117**	-0.11	Neg	0.20	Neg	0.09	Neg
**118**	-0.19	Neg	0.33	Neg	0.08	Neg
**119**	-0.24	Neg	0.26	Neg	0.09	Neg

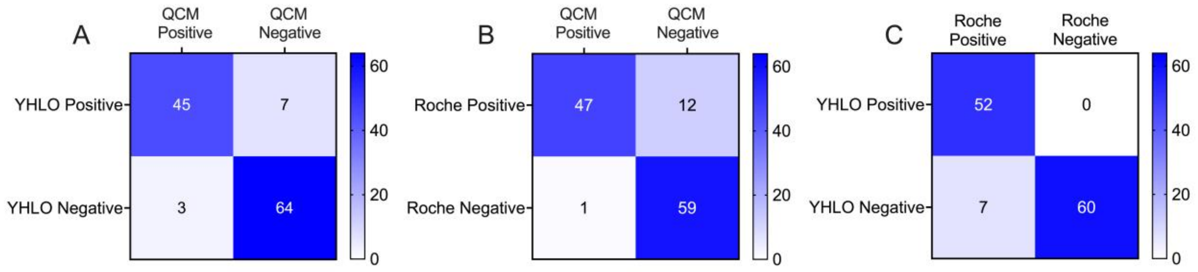

Figure S-4. Coherence between QCM, Roche, and YHLO SARS-CoV-2 antibody assays. Sera were analyzed for the presence of antibodies against SARS-CoV-2 RBD spike protein. Of the 119 analyzed samples, 48, 52, and 59 were positive in the QCM, Roche, and YHLO assays. The figure represents coherence between QCM and YHLO (A), QCM and Roche (B), Roche and YHLO (C).
